# Awareness of adapting clinical practice guidelines to a local context

**DOI:** 10.1002/gin2.70025

**Published:** 2025-05-06

**Authors:** Dawid Pieper, Eva‐Maria Behnke, Rieke Dumke, Christel Kehr‐Fuckel, Carolin Radow, Nils Schulze, Markus Follmann, Corinna Schaefer, Robert Prill, Christian Kopkow, Carolin Bahns, Kyung‐Eun (Anna) Choi, Lena Fischer

**Affiliations:** ^1^ Institute for Health Services and Health Systems Research, Brandenburg Medical School Theodor Fontane (MHB), Faculty of Health Sciences Brandenburg Immanuel Klinik Rüdersdorf Brandenburg Germany; ^2^ German Guideline Program in Oncology German Cancer Society Berlin Germany; ^3^ German Network for Health Literacy (DNGK e.V.) Cologne Germany; ^4^ Center of Orthopaedics and Traumatology University Hospital Brandenburg/Havel, Brandenburg Medical School Theodor Fontane Brandenburg Germany; ^5^ Faculty of Health Science Brandenburg Brandenburg an der Havel Brandenburg Germany; ^6^ Department of Therapy Science I Brandenburg University of Technology Cottbus‐Senftenberg Senftenberg Germany; ^7^ Centre for Health Services Research Brandenburg Medical School Theodor Fontane Neuruppin Germany; ^8^ Health Services Research Group, Research Centre Medical Image Analysis and Artificial Intelligence Danube Private University Krems‐Stein Austria

**Keywords:** clinical guidelines, Germany, guideline adaptation, locally adapted guidelines, survey

## Abstract

**Background:**

Guideline recommendations are often not implemented in practice. This can be attributed to factors such as patient preferences and characteristics, structural conditions, personnel or other resources, as well as cultural or ethical aspects. Adaptations to the local context (e.g., regional or hospital) result in so‐called locally adapted guidelines (LAGL), which could improve implementation. We aimed to assess the awareness of LAGL among guideline developers in Germany.

**Methods:**

An online survey was conducted via LimeSurvey in May 2024. The questionnaire, designed based on literature and expert opinions, consisted of 23 items, predominantly with dichotomous response options. Recruitment was conducted via email. Direct contact addresses were identified using the German guideline registry (*n* = 397). Additionally, a mailing list distribution was conducted through the guidelines working group of the German Network for Evidence‐Based Medicine (*n* = 316). Only fully completed questionnaires were included in the analysis. Data cleaning and descriptive analysis were performed using Excel.

**Results:**

A total of 63 questionnaires were fully completed. The most represented groups were physicians (65%) and methodologists (24%), most frequently in coordination (76%) or as group members (62%). The most judicious reasons for developing a LAGL were differences in patient populations (48%), currency of recommendations (46%), and patient values and preferences (44%). The most commonly cited likely reasons for developing a LAGL were economic considerations (32%), differences in patient populations (30%), and currency of recommendations (30%). Many respondents (59%) were aware of the possibility of adapting existing guidelines to the local context. Among these, approximately half (49%) had already locally adapted a guideline, with 75% using an adaptation framework.

**Discussion:**

LAGLs are known among guideline developers in Germany and are generally developed using adaptation frameworks. Potential reasons for preparing LAGLs are diverse, with some discrepancies between perceived valid and likely reasons.

## Highlights

- Over half (59%) of surveyed clinical practice guideline (CPG) developers in Germany were aware of the possibility of adapting CPGs to local contexts, and nearly half of them had practical experience doing so.
- Although several adaptation frameworks exist, only 44% of experienced adaptors were familiar with them, and among users, many found them only partly helpful.
- Differences in patient populations, outdated recommendations, and patient values were rated as the most valid reasons for local CPG adaptation, while economic factors were seen as the most likely driver in practice.
- Despite existing awareness, guideline adaptation to local settings remains underutilized and lacks structured implementation, highlighting the need for clearer definitions, better resources, and more practical examples.

## 1 INTRODUCTION

Although the importance of high quality clinical practice guidelines (CPGs) is widely acknowledged, adherence to guideline recommendations remain challenging.^1^ There are many potential reasons why CPGs fail to be implemented in practice.^2^ Discrepancies exist between CPGs and might depend on the clinical field, for example. Adherence to CPGs tends to vary depending on the recommendation. A well‐known barrier for implementation is the inconsistency between guideline recommendations and existing practice patterns. Lack of agreement with the recommendations due to lack of applicability or lack of evidence was the most perceived barrier by general practitioners in the Netherlands, for example.^3^ The local context can play a major role and be an important barrier or facilitator.^4^ Thus, knowledge about the local context is crucial for implementation strategies.^5^, ^6^ Tailoring guideline recommendations to the local context can help to overcome important barriers to use CPGs.^7^

Local contexts can differ due to differences in patients, personnel, structure, availability of resources, as well as cultural and ethical values.^8^, ^9^ Due to this, certain interventions might even become not feasible in practice.^10^ Thus, contextualization of CPGs and recommendations is critical. According to Schünemann et al., “contextualization […] describes the need for dialogue and formal consideration of the local best available evidence and criteria for adoption, adaption, or de novo creation of recommendations from an existing trustworthy guideline to a national, local, or other level. Considerations should be made to decide whether these recommendations are right for that setting and modifying or adding to the recommendations to optimize their implementation using structured and transparent processes”.^11^

CPG adaptation is mostly part of a de novo CPG development process.^12^ A scoping review found only examples of CPGs being adapted from one country to another.^13^ This can potentially save a lot of resources in terms of time and money when compared to de novo CPG development. There is a paucity of examples of CPGs that have been tailored a regional or local context. We acknowledge that the term ‘local context’ is currently ill‐defined. In the context of our study we mean any level below the national level by ‘local context’. This encompasses the tailoring of guideline recommendations to a state, region, and hospitals among others. Irrespective of the level, this process is also known as guideline adaptation. According to the Guidelines International Network (G‐I‐N) guideline adaptation is defined as “the systematic approach to the modification of a guideline(s) produced in one cultural and organizational setting for application in a different context”.

Many adaptation frameworks and guides have become available to support this process.^14^ While frameworks allow for formal adaptation, informal adaptation is also an option. According to a review that searched the literature until 2015, 60% of adapted CPGs did not use a formal adaptation method.^15^ To the best of our knowledge, the proportion of CPGs using any form of adaptation is unknown. However, the concept of adaptation seems to be underutilized in CPG development. There are many gaps in knowledge about CPG adaptation.^10^ There is a need for more research on how to best adapt CPGs to the local context.^16^ The aim of our study was to investigate the awareness of adapting CPGs to a local context in Germany.

## 2 METHODS

The survey is reported according to the checklist for reporting results of internet E‐surveys.^17^

### 2.1 Ethical approval and informed consent process

The study has received an ethics waiver by Brandenburg Medical School, Germany (Medizinische Hochschule Brandenburg, waiver no. 182022024‐ANF). Invitees were informed about the aim, the investigator, and the duration (approximately 10 min) of the survey. The survey was anonymous with the option of providing an email address for further inquiries. Invitees willing to participate had to confirm that they had carefully read the invitation email, and voluntarily agree to take the survey before access was granted.

### 2.2 Development and pretesting

In a first step, two authors (DP, LF) developed a draft of the questionnaire. The questionnaire was only prepared in German language. As we were not able to identify prior surveys on the same topic, the questionnaire was informed by literature on barriers and facilitators of CPGs as well as literature on CPG adaptation (frameworks). In a second step, the questionnaire was sent out for feedback to guideline developers, methodologists and health services researchers (MF, CS, CB, CK, AC). This included interest holders, specifically members of the German Agency for Quality in Medicine (CS), and the German Guideline Program in Oncology (MF). Both organizations are responsible for commissioning and supervising CPG development in Germany. In a last step, a final draft was prepared based on the received feedback in an iterative process including multiple rounds by members of the project team (DP, CKF, RD, NS, CR, EB). The final questionnaire was implemented in the online tool LimeSurvey. The same authors tested its applicability, usability, and technical functionality (including mobile devices) while determining the time needed to complete the survey. This was done informally without applying any benchmarks.

The final questionnaire contained 23 items with mostly closed questions (see Supporting Information file 1). It included questions on demographics, experience with CPGs, barriers and facilitators of CPGs, local adaptations, and adaptation frameworks. Respondents were able to go back to the previous pages. No item was mandatory.

### 2.3 Recruitment

We aimed to recruit CPG developers in Germany. To achieve this, we followed a two‐fold recruitment strategy. First, we searched the Register of the Association of Scientific Medical Societies in Germany for evidence and consensus based CPGs. In contrast to other international CPG classifications, the CPG system in Germany has a hierarchical structure with the following levels of CPGs: S1 (lowest level) recommendations by groups of experts, S2k Consensus‐based guidelines, S2e Evidence‐based guidelines, S3 Evidence‐ and consensus‐based guidelines characterized by a representative committee, systematic review and synthesis of the evidence and a structured consensus process. Thus, we only focused on S3 guidelines. The register includes all CPGs in medicine in Germany.^18^ We only searched for CPGs valid as of April 2024. For each CPG, we extracted the email address of the coordinating person as well as the CPG office. Second, we disseminated the link for the survey via the CPG working group of the German Network for Evidence‐based Medicine. Due to data protection rights, we were unable to obtain the contact data on our own as we did for the CPGs. All invitees were invited to share the survey link with other interested parties All respondents had to reply ‘yes’ to the first question asking whether they had ever participated in a CPG. In case ‘no’ was selected, the survey was terminated.

The survey was open from 16/5/2024 to 15/6/2024. A reminder was sent on 5/6/2024 to all invitees identified via the CPG register, irrespective of whether they had already taken the survey as the survey was anonymous. No incentives were provided for participation.

### 2.4 Data analysis

Data were automatically collected via LimeSurvey. We exported all collected data into Excel spreadsheets before data analysis. We only analyzed responses from individuals who completed the questionnaire. This choice was made post hoc due to a large number of people agreeing to participate by checking the checkbox but only filling the first items, if any. Quantitative data were analyzed descriptively with Microsoft Excel. We presented categorical data as proportions (percentage) and continuous data as median and interquartile range (IQR). Qualitative data were categorized where appropriate.

## 3 RESULTS

We identified 397 unique email addresses from 446 CPGs in the Register of the Association of Scientific Medical Societies in Germany. In addition, the survey link was shared with 316 members of the guideline working group of the German Network for evidence‐based medicine. We cannot rule out overlap in email addresses. Thus, the total number of invitees is not calculable.

Overall, 63 responses were analyzed. The demographic characteristics are shown in table 1. The respondents had a median age of 53.5 years, interquartile range (IQR) 44.25–59.5. Most respondents identified themselves as doctors (65%, 41/63) or methodologists (24%, 15/63). Most respondents were from internal medicine (19%, 12/63), general medicine (16%, 10/106), and pediatrics (16%, 10/106). Multiple answers were allowed for this question. More than three in four persons (76%) had acted at least once as a guideline coordinator/chair. The majority of respondents (78%, 49/63) indicated that they had held multiple roles in CPG development in their lifetime.

**Table 1:** Demographic characteristics.

Characteristic	*N* (%)
Gender (*n* = 63)	
Male	29 (46)
Female	29 (46)
Diverse	1 (2)
No answer	4 (6)
Number of CPGs worked on (*n* = 63)	
1 to 2	17 (27)
3 to 5	25 (40)
6 to 10	17 (27)
More than 10	4 (6)
Working experience with CPGs (*n* = 63)	
Less than 5 years	16 (25)
5 to less than 10 years	14 (22)
10 to less than 15 years	21 (33)
15 to less than 20 years	0 (0)
≥20 years	12 (19)
Profession (*n* = 63)	
Physician	41 (65)
Methodologist	15 (24)
Psychologist	1 (2)
Therapist	3 (5)
Other	3 (5)
Primary role in CPG development (*n* = 63)*	
Coordinator/panel chair	48 (76)
Steering group member	31 (49)
CPG group member	39 (62)
Methodologist	22 (35)
Other	5 (8)

*Note*: Acronym: CPG = clinical practice guideline; * Multiple answers allowed (*n* = 145 answers overall).

**Figure 1:**
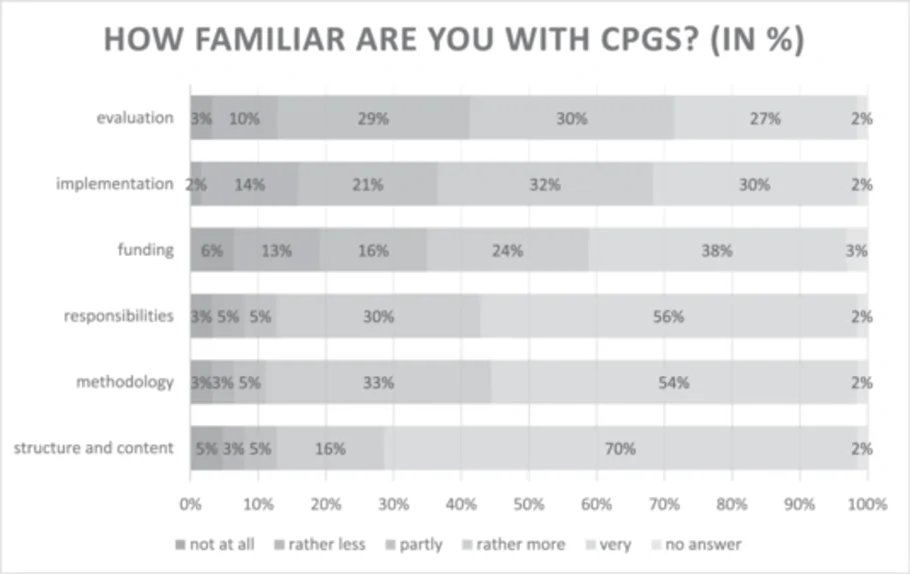
Familiarity with clinical practice guidelines (CPGs), *n* = 63.

Figure 1 shows how familiar the respondents felt with specific topics related to CPG development. The vast majority of respondents felt ‘rather more’ or ‘very’ familiar with the structure and content (86%, 54/63), methodology (87%, 55/63), and responsibilities of or within CPGs. Familiarity was lower with funding (62%, 37/63), implementation (62%, 37/63), and evaluation (57%, 36/63) of CPGs. Barriers to the following CPGs are provided in Supporting Information file 2.

**Figure 2:**
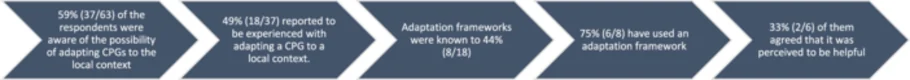
Awareness of adapting CPGs.

Overall, 59% (37/63) of the respondents were aware of the possibility of adapting CPGs to the local context. Of those, 49% (18/37) reported to be experienced with adapting a CPG to a local context. Adaptation frameworks were known to 44% (8/18) with GRADE‐ADOLOPMENT, Making GRADE the irresistible choice (MAGIC), and ADAPTE being the most commonly known adaptation frameworks (Supporting Information: file 3). Of these 8 persons, the majority (75%, 6/8) have used an adaptation framework, while only 33% (2/6) of them agreed that it was perceived to be helpful (figure 2). The remaining persons found it to be only partly helpful.

We also asked about potential reasons for adapting CPGs to a local text. The most ‘judicious’ reasons were differences in the patient population (i.e. actual patients differ from patients included in randomized trials used to derive recommendations), up‐to‐dateness of recommendations, and patients' values and preferences, with 48%, 46%, and 44% rating them as ‘rather more’ or ‘very’ judicious respectively. The most ‘likely’ reasons were found to be economic aspects (32%) followed by differences in the patient population (30%), and the up‐to‐dateness of recommendations (30%).

## 4 DISCUSSION

Our survey provides valuable insights about the awareness and actual use of adaptation frameworks in CPG development in Germany. More than half of the CPG developers were aware of the possibility of adapting CPGs to the local context. Approximately one in two of these had practical experience with it.

The respondents of our survey can be described as very experienced in developing CPGs. The vast majority of them participated in far more than one CPG. In a recently conducted survey on living guidelines in Germany, the mean number of CPGs in which respondents were a part of was 3.91.^19^ This is very much comparable to our sample. The same is also true for other characteristics except gender where we had an equal distribution. Notably, our sample size is lower than in former surveys in guideline developers in Germany.^19^, ^20^ We suspect there are two main reasons for this. Firstly, our study was conducted as part of a student project in the master's program on health services research at Brandenburg Medical School, Germany. Therefore, the time to carry out the survey and analyze data was limited to the summer term (i.e. April to July 2024). Some preparatory work had already been done beforehand. There were no resources for postal invitations and follow‐up calls as partly done in the other surveys. Secondly, the topic of our survey might be more specific and therefore attract less people. Respondents having heard of guideline adaptation before might have been more likely to take the survey compared to people never having heard about it before.

We are not aware of any studies we could directly compare our results to. However, the number of people being aware of guideline adaptation to a local context is higher than we expected. It is important to highlight that guideline adaptation is explicitly mentioned in the guidance document for preparing CPGs in Germany published by the Association of the Medical Societies.^21^ As all CPGs have to follow this guidance to be published in the corresponding CPG registry,^18^ one could have expected that guideline developers are aware of guideline adaptation in general. However, our survey explicitly focused on guideline adaptation to a local (i.e. subnational level) context. There is a risk that this was misunderstood by the respondents. This is against the background that we are not aware of many examples of guideline adaptations to a local context. Though some locally adapted guidelines have been evaluated in several countries,^22^, ^23^, ^24^, ^25^ we are unaware of such studies from Germany. We were also unable to identify any example of a locally adapted guideline from Germany. One possible explanation for our finding is that the respondents considered their hospital as the local context to which the original guideline was adapted. Adapting a guideline to one's own hospital is a frequent type of adapting to a local context.^25^, ^26^, ^27^ It can be expected that this is done often in practice without the usage of specific frameworks, and with limited evaluations or re‐audits. CPG recommendations could also serve as a basis for standard operating procedures (SOPs).^28^ They can even be seen as a way to bring evidence‐based recommendations into practice.^29^

When adaptation frameworks were used, they failed to be fully satisfactory. Challenges with using the adaptation frameworks in practice have been described by others.^12^, ^30^ Their application can be limited by the source guidelines due to methodological or reporting quality. Applying adaptation frameworks requires a high level of expertise and experience. Both resources may not be available in groups planning local adaptation. The process of adaptation is time‐consuming and complex, while methodological guidance mostly focuses on users with prior experience. GRADE‐ADOLOPMENT^13^ and ADAPTE^31^ have been described as the most widely used adaptation frameworks.^32^

Our survey found several reasons why adapting CPGs to the local context might be judicious, with differences in the patient population being the most meaningfully perceived reason. This is consistent with other surveys on barriers to using CPGs. For example, a survey conducted in Brazil found 57% of respondents agreeing that the ‘applicability of guidelines to a single patient’ was a barrier.^33^ This can have several reasons, with comorbid conditions being among the most important ones.^34^, ^35^ Economic aspects were found to be the most likely reason why CPGs would be adapted to the local context, while they were also found to be less judicious than other reasons. According to a recently published systematic review, financial aspects are very important in the context of the German healthcare system.^36^ Up‐to‐dateness of recommendations was also mentioned by many as a reason for guideline adaptation. Although this is a very important point, it is not specific to our study.

Our study has some limitations. Due to time constraints arising from the context of the study, there was no study protocol published a priori. The target population was not involved in technically testing the final questionnaire. We are not able to compute a response rate due to our recruitment process. The number of respondents is lower than in other national surveys with CPG developers, while our topic is more specific. As we only focused on Germany, our findings might not be generalizable to other countries.

Guideline developers still need to be better informed about the possibilities of guideline adaptation. This is likely to hold true for guideline adaptation in general as well as in the specific local context. As all guideline developers in Germany must follow the methodological guidance provided by the Association of Medical Societies, the corresponding section in the handbook could provide more details. Equally important is the provision of good examples of how guideline adaptation was done. Currently, there is no overview of adapted CPGs in Germany.

On an international level, more research on adapting CPGs specifically to a local context is needed. This must take into account that the term ‘local’ is currently ill‐defined. As such, “Adaptation to a local context” can encompass tailoring CPG recommendations to a regional healthcare system, to a group of healthcare providers or to single healthcare providers (e.g. hospitals). Furthermore, it is crucial that national experiences with guideline adaptation are shared internationally.

## AUTHOR CONTRIBUTIONS

Dawid Pieper; Methodology: Dawid Pieper, Eva‐Maria Behnke, Christel Kehr‐Fuckel, Rieke Dumke, Nils Schulze, Carolin Radow, Lena Fischer, Markus Follmann, Corinna Schaefer, Carolin Bahns, Christian Kopkow, Robert Prill, Kyung‐Eun (Anna) Choi; Software: Eva‐Maria Behnke; Validation: Dawid Pieper; Formal analysis: Dawid Pieper, Eva‐Maria Behnke, Christel Kehr‐Fuckel, Rieke Dumke, Nils Schulze, Carolin Radow; Investigation: Dawid Pieper, Eva‐Maria Behnke, Christel Kehr‐Fuckel, Rieke Dumke, Nils Schulze, Carolin Radow; Resources: Dawid Pieper, Rieke Dumke, Christel Kehr‐Fuckel, Nils Schulze; Data curation: Carolin Radow, Eva‐Maria Behnke; Writing – Original Draft: Dawid Pieper; Writing – Review & Editing: Dawid Pieper, Eva‐Maria Behnke, Christel Kehr‐Fuckel, Rieke Dumke, Nils Schulze, Carolin Radow, Carolin Bahns, Markus Follmann, Corinna Schaefer, Robert Prill, Kyung‐Eun (Anna) Choi, Christian Kopkow, Lena Fischer; Visualization: Eva‐Maria Behnke, Rieke Dumke, Dawid Pieper; Supervision: Dawid Pieper, Lena Fischer; Project administration: Dawid Pieper; Funding acquisition: not applicable.

## CONFLICT OF INTEREST STATEMENT

The authors declare no conflicts of interest.

## ETHICS STATEMENT

The need for an ethics approval was waived by Brandenburg Medical School, Germany.

## Supporting information

2025 02 24 supplementary file 1.

2024 12 06 Supplementary file 2.

Supplementary file 3.

## Data Availability

The data that support the findings of this study are available from the corresponding author upon reasonable request.
