# Supplementary material for: Awareness of adapting clinical practice guidelines to a local context
Source: Clin Public Health Guidel. 2025 May 6;2(3):e70025. doi: 10.1002/gin2.70025 (PMC13563397; doi:10.1002/gin2.70025)
Supplement: Supplementary file 1 [file GIN2-2-e70025-s001.docx]

Supplementary file 2:

reasons for not following CPG recommendations (in %)

|  | not at all | rather less | partly | rather more | very | no answer |
| --- | --- | --- | --- | --- | --- | --- |
| others' opinion personal opinion does not match CPG | 6.34921% | 9.52381% | 23.8095% | 28.5714% | 12.6984% | 19.0476% |
| others' opinion no integration in clinical system | 19.0476% | 14.2857% | 19.0476% | 22.2222% | 3.1746% | 22.2222% |
| others' opinion discordant CPGs (recommendations) | 12.6984% | 15.873% | 26.9841% | 14.2857% | 9.52381% | 20.6349% |
| others' opinion weak evidence | 7.93651% | 17.4603% | 25.3968% | 15.873% | 12.6984% | 20.6349% |
| others' opinion CPG is not up-to-date | 9.52381% | 12.6984% | 28.5714% | 12.6984% | 17.4603% | 19.0476% |
| others' opinion no availability/access to recommended therapies/procedures | 11.1111% | 14.2857% | 15.873% | 23.8095% | 14.2857% | 20.6349% |
| others' opinion lack of structure and processes | 7.93651% | 14.2857% | 26.9841% | 15.873% | 14.2857% | 20.6349% |
| others' opinion lack of time | 12.6984% | 25.3968% | 17.4603% | 15.873% | 9.52381% | 19.0476% |
| others' opinion CPG not sufficiently comprehensive | 22.2222% | 17.4603% | 20.6349% | 12.6984% | 7.93651% | 19.0476% |
| personal opinion personal opinion does not match CPG | 19.0476% | 17.4603% | 19.0476% | 9.52381% | 4.7619% | 30.1587% |
| personal opinion no integration in clinical system | 33.3333% | 12.6984% | 4.7619% | 14.2857% | 4.7619% | 30.1587% |
| personal opinion discordant CPGs (recommendations) | 22.2222% | 17.4603% | 12.6984% | 6.34921% | 11.1111% | 30.1587% |
| personal opinion weak evidence | 15.873% | 6.34921% | 14.2857% | 14.2857% | 20.6349% | 28.5714% |
| personal opinion CPG is not up-to-date | 14.2857% | 9.52381% | 17.4603% | 17.4603% | 14.2857% | 26.9841% |
| personal opinion no availability/access to recommended therapies/procedures | 23.8095% | 9.52381% | 7.93651% | 22.2222% | 9.52381% | 26.9841% |
| personal opinion lack of structure and processes | 19.0476% | 9.52381% | 25.3968% | 9.52381% | 9.52381% | 26.9841% |
| personal opinion lack of time | 33.3333% | 12.6984% | 12.6984% | 14.2857% | 0% | 26.9841% |
| personal opinion CPG not sufficiently comprehensive | 34.9206% | 14.2857% | 11.1111% | 9.52381% | 3.1746% | 26.9841% |
