# Supplementary material for: Awareness of adapting clinical practice guidelines to a local context
Source: Clin Public Health Guidel. 2025 May 6;2(3):e70025. doi: 10.1002/gin2.70025 (PMC13563397; doi:10.1002/gin2.70025)
Supplement: Supplementary file 2 [file GIN2-2-e70025-s002.docx]

supplementary file 1: questionnaire on locally adapted guidelines

**Dear members of clinical practice guideline groups,**

**Thank you for supporting our project on locally adapted guidelines in Germany with your time and expertise. Answering the following questions will take approximately 10 minutes. Should you wish to make changes to your answers, you have the option of navigating backward and forward within the survey using the “back” and “next” buttons provided (not via the browser).**

**On the last page of the questionnaire, you also have the opportunity to leave us your comments on the survey.**

**We hope you enjoy completing the questionnaire and would like to thank you once again for your participation!**

**Best regards,**

**The project team of the Brandenburg Medical School Theodor Fontane**

**Eva Behnke, Dr. Rieke Dumke, Christel Kehr-Fuckel, Prof. Dr. Dawid Pieper, Carolin Radow, Nils Schulze**

**Part A: locally adapted guidelines**

**A1. Have you ever participated in the development of a clinical practice guideline (CPG)?**

Yes	☐

No	☐

**A2. Your age**

**A3. Your gender**

Male	☐

Female	☐

Diverse	☐

No answer	☐

**A4. How many CPGs (including updates) have you been involved in?**

**A5. Please assign yourself to one of the participating groups.**

Physician	☐

Patient representative	☐

Patient	☐

Family member	☐

Nursing staff	☐

Therapist	☐

Psychologist	☐

Ethicist	☐

Methodologist	☐

No answer	☐

Other	☐

**A6. Please select the thematic areas of all the CPGs you are/have been involved in (multiple answers possible)**

General medicine	☐

Anesthesiology	☐

Ophthalmology	☐

Occupational medicine	☐

Surgery (all subspecialties)	☐

Chronic conditions	☐

Gynecology/Obstetrics	☐

Otorhinolaryngology	☐

Dermatology and venereology	☐

Hygiene and environmental medicine	☐

Infectious diseases	☐

Internal medicine	☐

Intensive and emergency medicine	☐

Pediatrics	☐

Neurology	☐

Oncology	☐

Orthopedics and trauma surgery	☐

Prevention, nutrition, and sports	☐

Psychiatry and psychotherapy	☐

(including child and adolescent psychiatry and psychotherapy)

Rare diseases	☐

Radiation therapy	☐

Urology	☐

No answer	☐

Other	☐

**A7. How long have you been working in your profession?**

< 5 years	☐

5 to < 10 years	☐

10 to < 15 years	☐

≥ 20 years	☐

**A8. How many years of experience do you have in developing CPGs?**

< 5 years	☐

5 to < 10 years	☐

10 to < 15 years	☐

≥ 20 years	☐

**A9. What role(s) did you play in the development of CPGs? (multiple answers possible)**

Coordinator	☐

Member of steering group	☐

Member of CPG group	☐

Methodologist	☐

No answer	☐

Other	☐

**A10. How did your participation in the CPG(s) come about? (multiple answers possible)**

Through a professional society/association	☐

Through a patient organization	☐

Through a research organization	☐

Through personal connections	☐

I don’t remember	☐

No answer	☐

Other	☐

**A11. In your opinion, how familiar are you with CPGs?**

**[1 = not at all; 2 = rather less; 3 = partly; 4 = rather more; 5 = very]**

|  | 1 | 2 | 3 | 4 | 5 |
| --- | --- | --- | --- | --- | --- |
| Structure and content | ☐ | ☐ | ☐ | ☐ | ☐ |
| Methodology | ☐ | ☐ | ☐ | ☐ | ☐ |
| Responsibilities | ☐ | ☐ | ☐ | ☐ | ☐ |
| Funding | ☐ | ☐ | ☐ | ☐ | ☐ |
| Implementation | ☐ | ☐ | ☐ | ☐ | ☐ |
| Evaluation | ☐ | ☐ | ☐ | ☐ | ☐ |

**A12. What reasons contribute to you or others not following CPG recommendations in your daily practice?**

**[1 = not at all; 2 = rather less; 3 = partly; 4 = rather more; 5 = very]**

**Others**

|  | 1 | 2 | 3 | 4 | 5 |
| --- | --- | --- | --- | --- | --- |
| The personal opinion is not in line with the CPG. | ☐ | ☐ | ☐ | ☐ | ☐ |
| Digital support systems do not integrate the CPG. | ☐ | ☐ | ☐ | ☐ | ☐ |
| Discordant CPGs/recommendations exist. | ☐ | ☐ | ☐ | ☐ | ☐ |
| The primary evidence is weak. | ☐ | ☐ | ☐ | ☐ | ☐ |
| The CPG is not up to date. | ☐ | ☐ | ☐ | ☐ | ☐ |
| The CPG is difficult to follow due to availability/accessibility of recommended procedures/therapies. | ☐ | ☐ | ☐ | ☐ | ☐ |
| The CPG is difficult to follow due to structural/processual reasons. | ☐ | ☐ | ☐ | ☐ | ☐ |
| The CPG is difficult to follow due to time constraints. | ☐ | ☐ | ☐ | ☐ | ☐ |
| The CPG is not sufficiently comprehensible. | ☐ | ☐ | ☐ | ☐ | ☐ |

**A13. What reasons contribute to you or others not following CPG recommendations in your daily practice?**

**[1 = not at all; 2 = rather less; 3 = partly; 4 = rather more; 5 = very]**

**I myself**

|  | 1 | 2 | 3 | 4 | 5 |
| --- | --- | --- | --- | --- | --- |
| The personal opinion is not in line with the CPG. | ☐ | ☐ | ☐ | ☐ | ☐ |
| Digital support systems do not integrate the CPG. | ☐ | ☐ | ☐ | ☐ | ☐ |
| Discordant CPGs/recommendations exist. | ☐ | ☐ | ☐ | ☐ | ☐ |
| The primary evidence is weak. | ☐ | ☐ | ☐ | ☐ | ☐ |
| The CPG is not up to date. | ☐ | ☐ | ☐ | ☐ | ☐ |
| The CPG is difficult to follow due to availability/accessibility of recommended procedures/therapies. | ☐ | ☐ | ☐ | ☐ | ☐ |
| The CPG is difficult to follow due to structural/processual reasons. | ☐ | ☐ | ☐ | ☐ | ☐ |
| The CPG is difficult to follow due to time constraints. | ☐ | ☐ | ☐ | ☐ | ☐ |
| The CPG is not sufficiently comprehensible. | ☐ | ☐ | ☐ | ☐ | ☐ |

**A14. Have you ever heard of the possibility of adapting an existing CPGs or its recommendations for a local context, e.g. for the needs of your own care practice?**

Yes	☐

No	☐

No answer	☐

**A15. Have you ever locally adapted a CPG?**

Yes	☐

No	☐

No answer	☐

**A16. There is indeed the possibility of adapting an existing CPG or its recommendations to a local context, e.g., for the needs of your own care practice. Based on this knowledge, we ask the following questions.**

I have taken note of this. ☐

**A17. How likely do you think it is that a CPG recommendation is adapted to the local context due to the following factors, for example, to meet the needs of your care practice?**

**[1 = not at all; 2 = rather less; 3 = partly; 4 = rather more; 5 = very]**

*If you select the option 'Other reason’, you will have the opportunity to enter your answer on the next page.*

|  | 1 | 2 | 3 | 4 | 5 |
| --- | --- | --- | --- | --- | --- |
| Disease frequency/prevalence or health status | ☐ | ☐ | ☐ | ☐ | ☐ |
| Differences in patient population | ☐ | ☐ | ☐ | ☐ | ☐ |
| Missing or additional technical equipment | ☐ | ☐ | ☐ | ☐ | ☐ |
| Missing or additional staffing resources | ☐ | ☐ | ☐ | ☐ | ☐ |
| Credibility/quality of the CPG | ☐ | ☐ | ☐ | ☐ | ☐ |
| Weak evidence base | ☐ | ☐ | ☐ | ☐ | ☐ |
| Up-to-dateness of recommendations | ☐ | ☐ | ☐ | ☐ | ☐ |
| Patient values and preferences | ☐ | ☐ | ☐ | ☐ | ☐ |
| Economic aspects | ☐ | ☐ | ☐ | ☐ | ☐ |
| Other reason | ☐ | ☐ | ☐ | ☐ | ☐ |

**A18. How judicious do you think it is that a CPG recommendation is adapted to the local context due to the following factors, for example, to meet the needs of your care practice?**

**[1 = not at all; 2 = rather less; 3 = partly; 4 = rather more; 5 = very]**

*If you select the option 'Other reason’, you will have the opportunity to enter your answer on the next page.*

|  | 1 | 2 | 3 | 4 | 5 |
| --- | --- | --- | --- | --- | --- |
| Disease frequency/prevalence or health status | ☐ | ☐ | ☐ | ☐ | ☐ |
| Differences in patient population | ☐ | ☐ | ☐ | ☐ | ☐ |
| Missing or additional technical equipment | ☐ | ☐ | ☐ | ☐ | ☐ |
| Missing or additional staffing resources | ☐ | ☐ | ☐ | ☐ | ☐ |
| Credibility/quality of the CPG | ☐ | ☐ | ☐ | ☐ | ☐ |
| Weak evidence base | ☐ | ☐ | ☐ | ☐ | ☐ |
| Up-to-dateness of recommendations | ☐ | ☐ | ☐ | ☐ | ☐ |
| Patient values and preferences | ☐ | ☐ | ☐ | ☐ | ☐ |
| Economic aspects | ☐ | ☐ | ☐ | ☐ | ☐ |
| Other reason | ☐ | ☐ | ☐ | ☐ | ☐ |

**A19. Other reason:**

**A20. “Adaptation Frameworks” can be helpful for adapting CPGs and/or recommendations. Have you heard of them before?**

Yes	☐

No	☐

No answer	☐

**A21. Which “Adaptation Frameworks” are you familiar with? (multiple answers possible)**

ADAPTE	☐

The Adapted ADAPTE by the Alexandria Center for Evidence-Based Clinical Practice Guidelines (EBCPG)	☐

Alberta Ambassador Program	☐

GRADE-ADOLOPMENT	☐

Making GRADE the Irresistible Choice (MAGIC)	☐

RAPADAPTE	☐

Royal College of Nursing (RCN)	☐

Systematic Guideline Review Method (SGR)	☐

No answer	☐

**A22. Have you already used an “Adaptation Framework”?**

Yes	☐

No	☐

No answer	☐

**A23. How helpful did you find the “Adaptation Framework”?**

**[1 = not at all; 2 = rather less; 3 = partly; 4 = rather more; 5 = very]**

1	☐

2	☐

3	☐

4	☐

5	☐

**A24. Thank you for your participation in our survey. If you have any further comments on this topic, we look forward to receiving your suggestions. Please use the following field to share your feedback with us.**

**Thank you for participating in this survey. We value your feedback and appreciate your time. If you have any further questions, feel free to contact us. Thank you and have a good day!**
