# Supplementary material for: Awareness of adapting clinical practice guidelines to a local context
Source: Clin Public Health Guidel. 2025 May 6;2(3):e70025. doi: 10.1002/gin2.70025 (PMC13563397; doi:10.1002/gin2.70025)
Supplement: Supplementary file 3 [file GIN2-2-e70025-s003.docx]

supplementary file 3: known adaptation frameworks (n=8 respondents)

| *Adaptation Frameworks* | *N (%)* |
| --- | --- |
| ADAPTE | 4 (50) |
| The Adapted ADAPTE by the Alexandria Center for Evidence-Based Clinical Practice Guidelines (EBCPG) | 1 (13) |
| Alberta Ambassador Program | 0 (0) |
| GRADE-ADOLOPMENT | 5 (63) |
| Making GRADE the Irresistible Choice (MAGIC) | 4 (50) |
| RAPADAPTE | 1 (13) |
| Royal College of Nursing (RCN) | 0 (0) |
| Systematic Guideline Review Method (SGR) | 1 (13) |
